# Investigating the biomarker potential and molecular targets of TIGD1 in lung cancer using bioinformatics

**DOI:** 10.55730/1300-0144.5920

**Published:** 2024-08-19

**Authors:** Merve Gülsen BAL ALBAYRAK, Tuğcan KORAK, Murat KASAP, Gürler AKPINAR

**Affiliations:** Department of Medical Biology, Faculty of Medicine, Kocaeli University, Kocaeli, Turkiye

**Keywords:** Lung cancer, biomarker, TIGD1, NSCLC, LUSC

## Abstract

**Background/aim:**

Lung cancer, a predominant contributor to cancer mortality, is characterized by diverse etiological factors, including tobacco smoking and genetic susceptibilities. Despite advancements, particularly in nonsmall-cell lung cancer (NSCLC), therapeutic options for lung squamous cell carcinoma (LUSC) are limited. Transposable elements (TEs) and their regulatory proteins, such as tigger transposable element derived (TIGD) family proteins, have been implicated in cancer development. TIGD1, upregulated in various cancers, including LUSC, lacks a defined function. The aim of our study was to elucidate the biological functions, associated pathways, and interacting proteins of TIGD1.

**Materials and methods:**

The GSE229260 microarray dataset was investigated using the GEO2R tool to identify the differentially expressed genes (DEGs) in TIGD1 silenced in A549 lung cancer cells in contrast to controls. Enrichment analyses and protein–protein interaction (PPI) network construction were performed to uncover key pathways using KEGG and STRING analyses. Hub genes were determined through the intersection of DEGs with lung cancer-related genes via Cytoscape software and the cytoHubba plug-in, and their functions were analyzed. Immune and stromal scores of hub genes were also evaluated using the ESTIMATE algorithm.

**Results:**

Analyzing microarray data from TIGD1-silenced A549 NSCLC cells, a total of 13 upregulated DEGs and 1 downregulated DEGs were identified. The TIGD1-associated DEGs revealed significant involvement in crucial molecular pathways, including the PI3K/AKT, FOXO, and p53 signaling pathways. The hub genes AKT1, BRAF, SRC, GAPDH, CCND1, CDKN2A, CTNNB1, KRAS, MYC, and TP53 emerged as central regulators of cell proliferation, apoptosis, and protein metabolism. The hub genes exhibited negative correlations with immune and stromal components in the tumor microenvironment, suggesting their potential as biomarkers for lung cancer therapy.

**Conclusion:**

This study elucidates the potential functions of TIGD1 in lung cancer and identifies promising biomarker candidates associated with TIGD1 gene expression, presenting potential therapeutic targets for lung cancer therapies.

## 1. Introduction

Cancer, a significant global public health challenge, is characterized by dysregulated cellular proliferation, giving rise to malignant neoplasms and distant metastasis [1]. It arises from genetic mutations that disturb the regulatory mechanisms of the cells responsible for cell division, apoptosis, and DNA repair [2]. Lung cancer, despite technological progress, continues to predominate as the leading cause of global cancer mortality [3]. GLOBOCAN 2018 estimates surpassed 2 million new cases and 1.8 million deaths, underscoring increasing incidence and fatality [4]. Prevalent as the primary cause of cancer-related deaths in males and secondary in females, lung cancer’s etiology is principally linked to tobacco smoking, while additional determinants such as lung infections, environmental exposure to asbestos or radon, and genetic susceptibilities intricately contribute to its pathogenesis [5].

Lung cancer is predominantly categorized into two primary types: nonsmall-cell lung cancer (NSCLC), representing approximately 85% of cases, and small-cell lung cancer (SCLC), accounting for the remaining 15% [6]. NSCLC is further stratified into lung adenocarcinoma (LUAD), lung squamous cell carcinoma (LUSC), and large cell carcinoma (LCC) [7]. These subtypes exhibit distinctions in their cellular origins and underlying molecular and cellular mechanisms, influencing their tendency for distant metastasis and sensitivity to therapeutic agents. Notably, SCLC and LUSC are particularly prevalent in smokers. LUSC, accounting for 40% of all lung cancers, currently lacks targeted agents. Therefore, it is imperative to explore potential molecular targets for enhancing LUSC therapies.

Transposable elements (TEs), present in the genomes of nearly all organisms, possess the capacity to reposition themselves and undergo replication. This capability can lead to changes in gene expressions and the generation or reversal of mutations [8]. Regulation of TEs plays a pivotal role in cancer development by influencing alterations in gene expressions [9–15]. The tigger transposable element-derived (TIGD) family proteins appear to be related to the DNA transposons and exhibit homology to centromere protein B (CENP-B), a factor previously associated with various cancers [16]. TIGD1, a member of the TIGD family of proteins, demonstrates upregulation in eight distinct human cancers, including LUSC. Its function is currently unknown [17]. In a study investigating the roles of TIGD1 in oral squamous cell carcinoma (OSCC), it was revealed that the expression of the gene is associated with tumor infiltration [16]. In another study, TIGD1 was identified as a novel oncogene in the progression of colon cancer [18]. In the study of colorectal cancer (CRC), it is suggested that TIGD1 acts as a regulatory protein in the process of cuproptosis in CRC cells [19]. These studies suggest that TIGD1 potentially plays a role in the progression and development of cancer. However, to elucidate the biological functions and associated pathways of the TIGD1 protein, further investigations are required.

The GSE229260 dataset, obtained from the Gene Expression Omnibus (GEO) database, includes data where TIGD1 was silenced using specific small-hairpin RNA (shRNA) in A549 lung cancer cells, a cell line representative of the NSCLC subtype. This dataset illustrates the functional role of TIGD1 in lung cancer by analyzing the expression pattern changes resulting from TIGD1 knockdown using the Human Gene Expression Array. Subsequently, the downstream effects and potential molecular targets associated with TIGD1 silencing in NSCLC were investigated.

The objective of the present study was to explore the biological functions, associated pathways, and interacting proteins of TIGD1. To achieve this, we acquired the GSE229260 microarray dataset and identified differentially expressed genes (DEGs) through bioinformatic algorithms, followed by enrichment analyses. Hub genes were subsequently discerned by intersecting DEGs with the PPI network of lung cancer. Following the enrichment and pathway analyses, the immune and stromal scores of the hub genes were revealed. Through these investigations, potential proteins interacting with TIGD1 and molecular pathways elucidating the functional role of the TIGD1 gene in lung cancer were determined.

## 2. Materials and methods

### 2.1. Data processing

Gene expression profiles from the dataset GSE229260 were acquired from the GEO database, a repository provided by the National Center for Biotechnology Information (NCBI). The data retrieval was conducted through the specified accession link.^1^ The dataset presented comprehensive expression profiles of A549 cells under both control conditions and conditions involving the inhibition of TIGD1 gene expression. Firstly, TIGD1 expression across various cancer types was analyzed using data from the TCGA database via the UALCAN platform. Then the samples used in the GSE229260 dataset underwent normalization, as evidenced by the generated boxplot using the GEO2R web tool.^2^ Identification of DEGs between the shControl and shTIGD1 groups was executed using the limma algorithm implemented in the R programming language through GEO2R by applying Benjamini–Hochberg false discovery rate adjustment. The threshold criteria for filtering were established as |logFC| ≥ 1.5 and an adjusted p-value less than 0.05 within the GSE229260 dataset. Utilizing identified DEGs, a volcano plot was generated through GEO2R. The expression levels of the identified DEGs were subjected to balloon plot analysis and hierarchical heatmap analysis, elucidating the divergences in expression patterns across distinct samples and groups using a public web tool, namely SRplot.^3^

### 2.2. Analysis of DEGs

To investigate protein–protein interactions, biological functions, and integrated molecular pathways, as well as diseases or gene ontologies associated with the DEGs, the identified common DEGs underwent Gene Ontology (GO) and Kyoto Encyclopedia of Genes and Genomes (KEGG) pathway enrichment analyses. These analyses were conducted using the online tool provided by the Search Tool for the Retrieval of Interacting Genes/Proteins (STRING).^4^ The STRING search parameters were set to an interaction score of medium confidence level (0.400), a maximum of ten interactors for both the first and second shells, and active interaction sources including text mining, experiments, databases, coexpression, neighborhood, gene fusion, and cooccurrence. The false discovery rates (FDRs) of hits considered in this analysis were below 1e−04. The resultant data were downloaded as bitmap images, and Adobe Illustrator Version 6 was used to generate the recreated images. Default parameters were applied, encompassing GO terms related to biological process (BP), cellular component (CC), and molecular function (MF), along with KEGG pathways with the threshold of p < 0.05. The enrichment bubble plots of the identified BP, CC, MF, and KEGG results were generated using SRPlot.

### 2.3. Identification of hub genes

The PPI network involving DEGs and their association with lung cancer was established using Cytoscape v3.10.1 software, incorporating the cytoHubba plug-in interfaced with the STRING database. The degree scores of the initial 100 genes within the network were scrutinized using a cutoff criterion of a required confidence (combined score) > 0.4. Following this, the generated PPI networks underwent intersection to reveal shared interactors. The recognition of the foremost 10 hub genes within the merged PPI was accomplished through the application of the maximal clique centrality (MCC) algorithm, a feature within the cytoHubba plug-in. Created images were exported as bitmap images, and Adobe Illustrator Version 6 was used to generate the recreated images. Pathway analysis was executed utilizing the enlisted hub genes and the lung cancer pathway from the KEGG pathway database (accession number: hsa05223), employing the pathway map within SRPlot.

### 2.4. Immune stromal and immune cell scores of the hub genes

The SangerBox 3.0 platform^5^ was used to obtain immune stromal and immune cell scores in lung cancer using the ESTIMATE (Estimation of STromal and Immune cells in MAlignant Tumor tissues using Expression) algorithm. The unified and standardized pan-cancer dataset from the UCSC database^6^ that specifically includes the TCGA TARGET GTEx dataset (PANCAN, N = 19,131, G = 60,499) was used. The gene expression data of each hub gene in each sample were extracted and transformed by log2 (x + 0.001). The R software package psych (version 2.1.6) (“corr.test” function) was utilized to calculate the Pearson’s correlation coefficient between the gene of interest and immune infiltration scores in lung cancer. Subsequently, the association was established between immune and stromal components in the tumor microenvironment (TME) and the expression levels of the hub genes (p-value < 0.05, |R| > 0.3).

## 3. Results

### 3.1. Identification of DEGs in lung cancer

**Figure 1:**
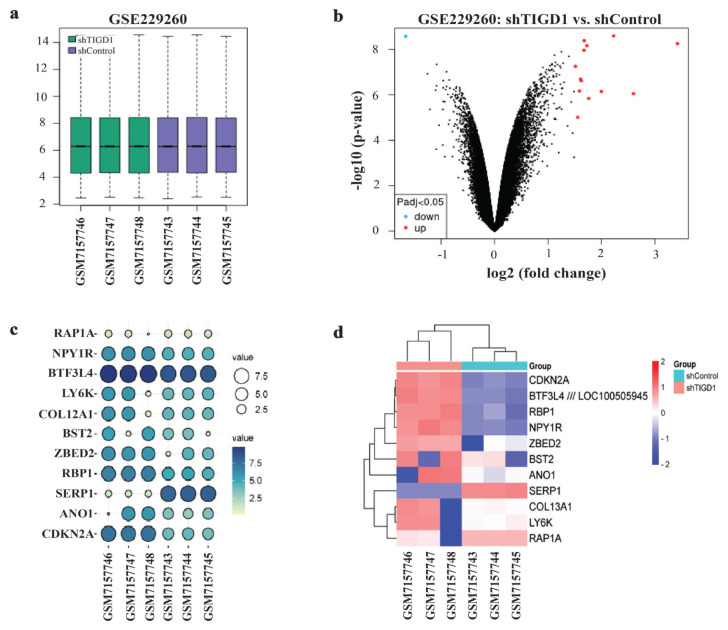
Analysis of GSE229260A and identification of differentially expressed genes (DEGs). **a.** Boxplot for gene expression of each sample in GSE229260 **b.** Volcano plot of GSE229260 and identification of DEGs, the cut-off criteria were |logFC| ≥ 1.5 and adjusted p < 0.05 in the GSE229260 dataset. Red, upregulated DEGs; Blue, downregulated DEGs. **c.** A balloon plot was generated to depict the relative abundance of DEG in GSE229260 dataset samples. The color and size of the circles within the plot correspond to the expression levels of the specified genes. **d.** Heatmap analysis was conducted to visualize the relative abundances of proteins in two distinct groups, namely shTIGD1 and shControl, within A549 cells. The heatmap illustrates the expression patterns of 10 significantly regulated proteins. The x-axis represents samples and the y-axis represents genes. The red blocks represent the overexpressed proteins, while the blue blocks represent altered proteins with the lowest expression levels, presented in a logarithmic scale ranging from −2 to 2.

TIGD1 expression analysis indicated that in most tumor types, including LUSC, the expression level was elevated compared to that in control samples (Supplementary Figure). In the analysis of the GSE229260, six samples were subjected to normalization, categorically assigned to two groups: shControl and shTIGD1. The boxplot reveals that the medians of the samples are centrally positioned and exhibit uniform levels. Furthermore, the interquartile range (IQR) and the width of the boxes indicate similar data dispersion across the samples. This suggests a high degree of uniformity across the dataset, indicating a stable and reproducible experimental outcome (Figure 1a). Subsequently, 14 genes were identified as exhibiting differential regulation. Thirteen of the DEGs were upregulated, whereas 1 of them was downregulated. Three of the DEGs were recognized as repetitive and consequently excluded from the analysis. The volcano plot illustrating the identified DEGs is depicted in Figure 1b. Differentially expressed genes were identified using Benjamini–Hochberg false discovery rate statistics, with the fold change and adjusted p-value documented in Table. Furthermore, Figure 1c illustrates the expression levels of the identified DEGs, highlighting consistent expression within samples of the same groups but variations between different groups. Furthermore, the expression profile of the commonly identified DEGs across all samples was systematically constructed and underwent hierarchical clustering. As illustrated in Figure 1d, samples treated with shControl (depicted in red) and shTIGD1 (depicted in blue) can be unequivocally categorized into two distinct subgroups.

### 3.2. Pathway analysis of identified DEGs

**Figure 2:**
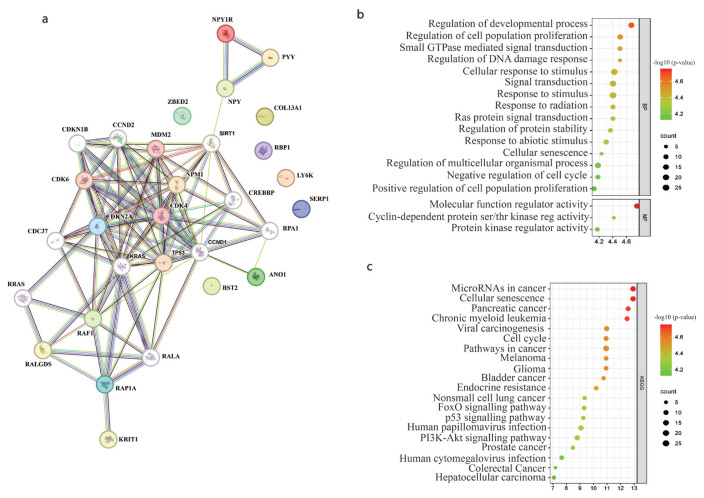
The functional and pathway enrichment analyses of the differentially regulated genes (DEGs). **a.** STRING analysis of DEGs, line thickness indicates the strength of data support, **b.** Bubble plots of Gene Ontology (GO) and **c.** Kyoto Encyclopedia of Genes and Genomes (KEGG) pathway enrichment analyses. The circumference of the circle corresponds to the gene count, the circle’s color represents the p-values, and the GeneRatio along the bottom transverse coordinate indicates the proportion relative to the total number of genes. (ser/thr: serine/threonine; reg: regulator.)

Analysis of the protein–protein interactions and regulated pathways between DEGs was performed using STRING analysis (Figure 2a) and GO and KEGG pathways were annotated. GO analysis indicated that prominent GO terms included “regulation of developmental process” (biological process category) and “molecular function regulator activity” (molecular function category) (see Figure 2b). Furthermore, KEGG pathway analysis revealed that the foremost enriched pathway was “microRNAs in cancer” (refer to Figure 2c).

### 3.3. Identification and analyses of the hub genes

**Figure 3:**
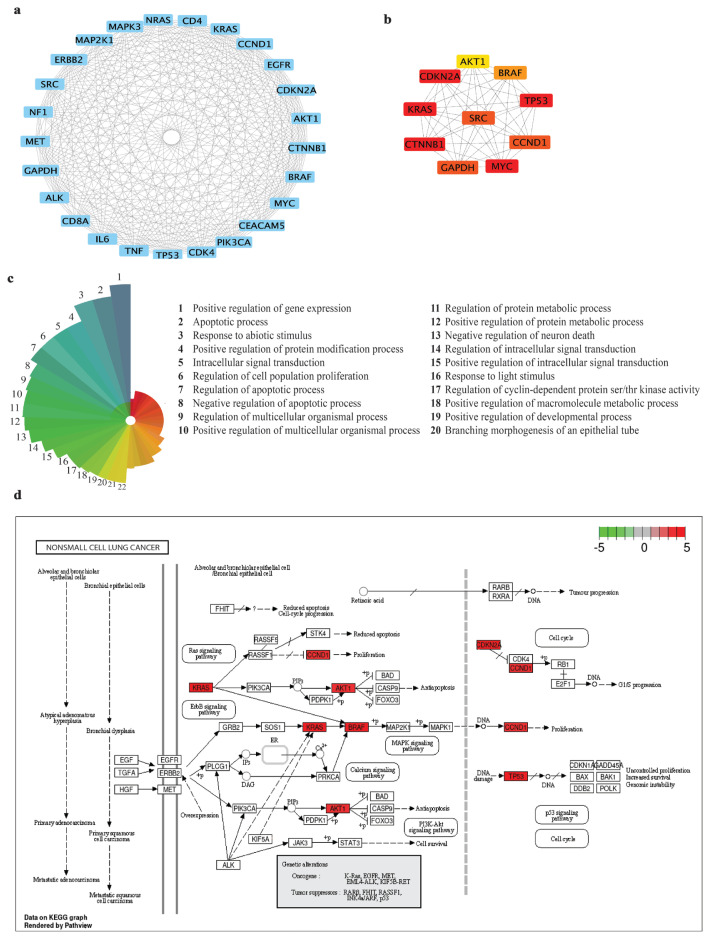
Identification and pathway analysis of the hub genes **a.** Visualization of the protein–protein interaction (PPI) network. The PPI network comprises the 25 overlapping genes, where blue nodes represent individual genes and edges depict interactions between them. **b.** Hub genes were generated from the PPI network using the maximal clique centrality (MCC) algorithm. Protein–protein associations are illustrated by edges, with red nodes denoting genes possessing high MCC scores and yellow nodes indicating genes with low MCC scores. **c.** Primary functions of hub genes were determined by ranking gene functionalities based on the prevalence of hub gene inclusion. **d.** Visualization of hub genes on KEGG database nonsmall cell lung cancer (hsa05223); the red color indicates the high expression levels of the hub genes in the pathway. The gene expression levels are calculated by the log fold change in the shTIGD1 group relative to the shControl group.

Using STRING analysis and the cytoHubba plug-in, the key genes within the PPI network were discerned through the intersection of lung cancer-related genes and DEGs. A total of 25 genes were identified from this intersection, as illustrated in Figure 3a. Subsequently, the MCC algorithm within the cytoHubba plug-in in Cytoscape was used to pinpoint hub genes within the PPI network, as depicted in Figure 3b. Among the 25 genes, the top 10 hub genes were determined to be TP53 (tumor protein p53), CTNNB1 (catenin beta 1), KRAS (KRAS proto-oncogene, GTPase), BRAF (B-Raf proto-oncogene, serine/threonine kinase), CCND1 (cyclin D1), GAPDH (glyceraldehyde-3-phosphate dehydrogenase), SRC (SRC proto-oncogene, nonreceptor tyrosine kinase), MYC (MYC proto-oncogene, bHLH transcription factor), CDKN2A (cyclin-dependent kinase inhibitor 2A), and AKT1 (AKT serine/threonine kinase 1). CDKN2A, identified as one of the DEGs, was notably upregulated in lung cancer-related genes, highlighting its specific and pivotal role in this context. The main functions of the hub genes are represented in Figure 3c ranged by the number of hub genes involved. Regulation of cellular apoptosis and proliferation were the primary functions of the hub genes. The enriched expression of the hub genes implicated in the NSCLC network was mapped by employing KEGG pathway maps through the Pathview tool in R facilitated by SRplot (refer to Figure 3d). Notably, all hub genes exhibited upregulated expression, denoted by the red coloration in Figure 3d.

### 3.4. Association of hub genes with immune cells

**Figure 4:**
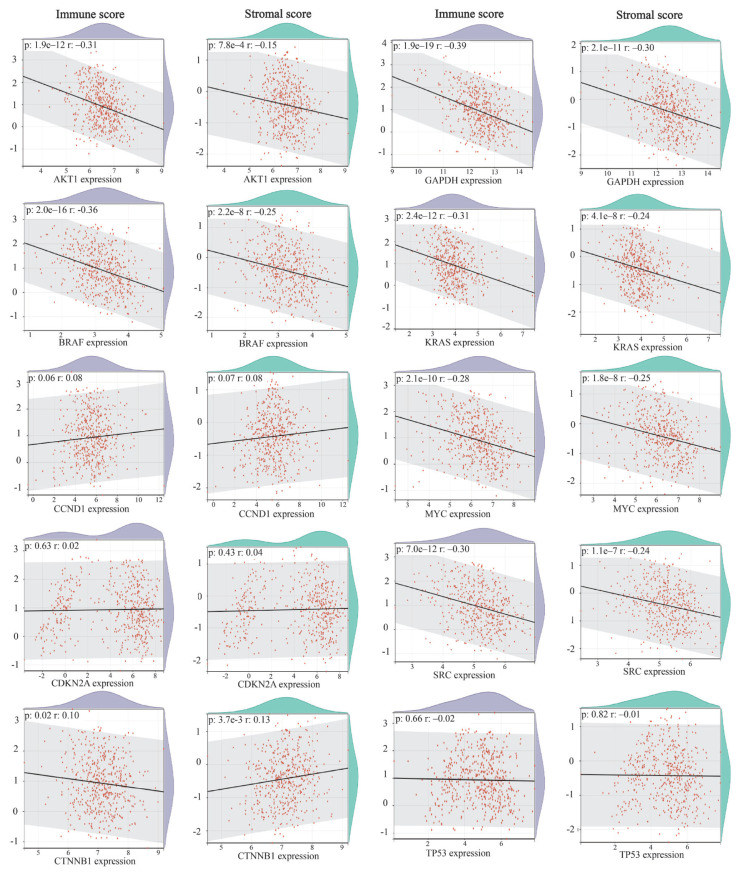
The association between the expression levels of hub genes and the immune score and stromal score in lung cancer.

Additionally, immune scores and stromal scores of the hub genes were measured (graphs shown in Figure 4). The immune score reflects the abundance of immune cells in the tumor microenvironment, while the stromal score indicates the presence of stromal cells. Both in the immune scores and in stromal scores, seven out of the 10 hub genes indicated significant correlation with lung cancer. In AKT1 (r = −0.31, p < 0.05), BRAF (r = −0.36, p < 0.05), CTNNB1 (r = −0.1, p < 0.05), GAPDH (r = −0.39, p < 0.05), KRAS (r = −0.31, p < 0.05), MYC (r = −0.28, p < 0.05), and SRC (r = −0.3, p < 0.05), immune score was negatively correlated with lung cancer and the highest correlation coefficient was in GAPDH. In AKT1 (r = −0.15, p < 0.05), BRAF (r = −0.25, p < 0.05), GAPDH (r = −0.3, p < 0.05), KRAS (r = −0.24, p < 0.05), MYC (r = −0.25, p < 0.05), and SRC (r = −0.24, p < 0.05), stromal score was negatively correlated with lung cancer and the highest correlation coefficient was in GAPDH. Stromal score was positively correlated with lung cancer expression in CTNNB1 (r = 0.13, p < 0.05). According to the results, the immune and stromal scores were consistent in all hub genes.

## 4. Discussion

Lung cancer is responsible for most cancer-related deaths, exhibiting a poor prognosis [20]. Despite several ongoing research efforts, there is a need to identify new prognostic biomarkers and therapeutic targets for lung cancer [21]. Consistent with our findings, TIGD1 is an overexpressed protein across various cancer types, including LUSC, but its function, associated proteins, and cellular mechanisms remain largely unknown [17]. In the present study, we analyzed the GSE229260 dataset from the GEO database, contrasting three samples treated with shControl against three samples with shTIGD1 in A549 LUSC cells. Our objective was to identify alterations in proteins or pathways arising from TIGD1 silencing and to elucidate the inherent function of the protein. In this analysis, we merged the protein–protein interaction network of identified DEGs with genes associated with lung cancer obtained from the TCGA-LUSC protein–protein interaction network, revealing hub genes. Furthermore, we conducted enrichment analysis and assessed the immune and stromal scores of the identified hub genes.

Differentially expressed genes were acquired and expression levels of the genes in the samples were presented in a balloon plot. Even though most of the genes show within-group consistence, the identification of certain genes displaying inconsistency among samples within the groups introduces a layer of complexity, underscoring the heterogeneous nature of individual gene responses to the treatments. The hierarchical clustering analysis reveals a clear and unequivocal categorization of the samples into two distinct subgroups. The clear relationships visualized between samples emphasize the reliability of the clustering results.

The functional enrichment analysis of the DEGs has unveiled associations with cellular regulatory mechanisms linked to developmental processes, cell population proliferation, and cellular response to stimulus and radiation, suggesting the potential involvement of these DEGs in shaping fundamental biological pathways in gene ontology fundamental biological processes. This offers insights into the multifaceted roles of DEGs associated with TIGD1 expression in both developmental and cancer contexts.

In the KEGG analysis, the identification of DEGs associated with microRNAs in cancer adds complexity to the findings, hinting at their potential implication in cancer biology. These results lay the groundwork for comprehensive investigations into the intricate molecular connections between DEGs and microRNA-mediated regulatory networks. Furthermore, cellular senescence, the phosphatidylinositol 3-kinase (PI3K)/protein kinase B (AKT) pathway, the p53 signaling pathway, and the FoxO signaling pathway were also found to be associated with the DEGs in the KEGG analysis. Carcinogenesis arises when the balance between cellular growth and death mechanisms is disrupted [22]. The PI3K/AKT pathway is a mechanism involved in cell survival, cell cycle progression, and cellular growth with key players PI3K, AKT, and MTOR. The pathway is upregulated in many tumors and plays a major role in tumor development [22]. The FOXO transcription factor family, with tumor-suppressive functions in various cancers, is involved in apoptosis, triggering the expression of death receptor ligands like Fas ligand, TNF apoptosis ligand and Bcl-XL, bNIP3, and Bim from Bcl-2 family members. The most important pathway interacting with FOXO in different types of cancers is the PI3K/AKT pathway [23]. Conversely, the p53 signaling pathway, critical for maintaining cellular homeostasis in healthy cells, is frequently mutated in human cancers [24]. Additionally, cellular senescence, a state of permanent cell cycle arrest in proliferating cells, acts as a potent barrier against tumorigenesis in cancer [25]. Given the involvement of the identified TIGD1-associated DEGs in pathways significant for the development of cancer cells, these DEGs show promise as potential biomarker candidates for lung cancer therapy. Their multifaceted roles in regulatory networks associated with both developmental processes and cancer biology make them valuable targets for further exploration in therapeutic interventions.

From the intersection of identified differentially expressed genes and the TCGA-LUSC PPI network, the top 10 hub genes were acquired utilizing the MCC algorithm. CDKN2A, CTNNB1, KRAS, MYC, and TP53 stand out as core genes within the hub genes (marked in red in Figure 3b). Among these, KRAS, MYC, and TP53 are considered well-known and frequently studied oncogenes. TP53, the most frequently mutated gene, functions as a tumor suppressor, with loss-of-function mutations predominantly observed. Interestingly, TP53 is also recognized as an oncogene, particularly with gain-of-function mutations contributing to tumor growth. This dual role underscores the complexity of TP53’s function and de novo transforming abilities, warranting further investigation. Specifically, in the context of lung cancers, TP53 mutations are most frequently observed in LUSC among the other types of NSCLC tumors. Additionally, abnormal TP53 status stands out as an indicator of shorter survival in LUSC and is proposed as a prognostic marker [26,27]. Conversely, KRAS is a well-established oncogene, intimately involved in carcinogenesis in most cancers, including lung cancer [28,29]. MYC, in addition to its oncogenic role in carcinogenesis, plays a pivotal role in the transcriptional program of healthy cells [30]. While these genes individually contribute to cancer development, their collective interplay and interactions with counterparts also play a significant role [31]. Understanding the intricate relationships between these proteins is crucial for unraveling the comprehensive molecular mechanisms driving carcinogenesis.

Exon 3 mutations in the CTNNB1 gene, responsible for encoding the β-catenin protein, have been identified in association with several cancers. These mutations lead to the reprogramming of the nuclear transcriptional network and an increase in cell proliferation [32]. Furthermore, somatic mutations in TP53 and CTNNB genes have been widely recognized as cancer drivers, particularly in hepatocellular carcinoma, occurring at a high frequency [33]. Notably, CDKN2A emerges as a promising candidate for a lung cancer biomarker, serving as an intersecting gene between differentially expressed genes and hub genes. Somatic mutations in CDKN2A, which encodes the cell-cycle inhibitor p16, have been observed in numerous cancers, establishing its role as a tumor suppressor [34]. Alterations in this gene have been reported to be dependent on the stage and site of NSCLC, often manifesting as homozygous deletions in either exon 1 or exon 2 [35,36]. In summary, the genes associated with TIGD1 function may serve as key players in the development of lung cancer and show potential as targets for lung cancer-targeted therapies. The identified genetic alterations, especially in CTNNB1, TP53, and CDKN2A, underscore their significance in the intricate molecular landscape of lung cancer progression.

Through enrichment analysis using the KEGG pathway, these hub genes emerged as key regulators of apoptosis, proliferation processes, and protein metabolic processes within the cellular context. The KEGG pathway map specific to NSCLC prominently illustrates the involvement of these hub genes in inducing proliferation, reducing apoptosis, and maintaining cell cycle progression—facilitating cancer cell growth.

In our analysis, we intersected the hub genes related to TIGD1 with the NSCLC pathway. Key hub genes, including KRAS, BRAF, CCND1, CDKN2A, TP53, and AKT1, emerged as central figures within these pathways. These genes are involved in crucial signaling mechanisms: the ERBB signaling pathway, which influences cell proliferation and survival [37]; the RAS signaling pathway, which affects cell growth and differentiation [38]; the p53 signaling pathway, which is pivotal for DNA repair and apoptosis [39]; and the cell cycle pathway, which regulates cell division. Specifically, KRAS and BRAF are prominent in both the ERBB and RAS signaling pathways, while CCND1 and CDKN2A are vital for cell cycle control. TP53 plays a dual role as a tumor suppressor and potential oncogene, particularly with gain-of-function mutations contributing to tumor progression. Additionally, TP53 mutations are most frequently observed in LUSC among the types of NSCLC, and its abnormal status is an indicator of shorter survival, making it a proposed prognostic marker [40]. AKT1 is a key player in the PI3K/AKT pathway, impacting cell survival and proliferation [41]. TIGD1 appears to be a critical regulator in NSCLC by modulating pathways and genes essential for cell proliferation, survival, and genomic integrity. Its influence on ERBB, RAS, p53 signaling, and cell cycle pathways suggests that TIGD1 could either facilitate tumor progression or act as a tumor suppressor, depending on the context. These pathways and their associated genes underscore the multifaceted role of TIGD1 in NSCLC, highlighting its potential as a therapeutic target. Understanding TIGD1’s precise regulatory mechanisms could reveal new therapeutic targets and strategies for lung cancer treatment, emphasizing the need for further studies to validate these findings in diverse models and clinical settings.

The tumor microenvironment comprises a dynamic interplay among immune, stromal, and tumor cells, exerting a pivotal influence on tumor prognosis. The composition of immune and stromal cells is intricately linked to patient survival, given the direct impact of immune cells on tumor cells [42,43]. Elevated immune and stromal scores signify heightened immune cell activity within the TME, correlating positively with both overall survival (OS) and progression-free interval (PFI) [44]. Similarly, it was observed that, in contrast to the low immune score group, patients with lung cancer in the medium and high immune score groups exhibited significantly extended disease-free survival (DFS) and OS. The findings suggest that the immune score might outperform the TNM staging system in predicting the DFS of lung cancer patients [45]. Therefore, a comprehensive analysis of TME components shows significant promise for the development of targeted drugs in tumor immunotherapy. Notably, our study identified a negative correlation between the expression of key hub genes (AKT, BRAF, GAPDH, KRAS, MYC, and SRC) and immune and stromal cell content in the lung cancer TME. Furthermore, the CTNNB1 gene exhibited a negative correlation with immune score but a positive correlation with stromal score, indicating its role in TME construction. These findings suggest a potential association between the identified genes and TME purity, influencing cancer aggressiveness and patient survival. Consequently, these genes may serve as promising targets to modulate OS, providing new avenues for therapeutic advancements in lung cancer treatment.

TIGD1-associated DEGs demonstrated a significant role in the regulation of cell proliferation and apoptosis, facilitating carcinogenesis. The analysis of hub genes adds a layer to the data, extending from NSCLC to lung cancer in general. Their functions parallel those of DEGs, encompassing the regulation of apoptosis and proliferation, with the additional involvement in protein metabolic processes. Moreover, the assessment of immune and stromal scores of the hub genes reflects the expression levels of genes crucial for lung cancer development. This multidimensional analysis further underscores the significance of these genes in the complex molecular landscape associated with lung cancer. The genes highlighted in the present study, whether identified as DEGs or hub genes, emerge as potential biomarker candidates for lung cancer therapies. Their multifaceted roles in key regulatory processes, as well as their impact on immune and stromal components, suggest their importance in understanding and targeting the intricacies of lung cancer progression.

Our study is based on a single dataset and a limited number of samples, primarily focusing on A549 lung cancer cells, which may not capture the full heterogeneity of lung cancer across different cell lines and patient samples. Although bioinformatic analyses identified key pathways, hub genes, and dynamics within the TME, functional validation in the context of lung cancer progression and therapy response was not conducted. To address these limitations, future studies should integrate diverse datasets, conduct in vivo validation using animal models, and perform functional experiments to corroborate the roles of identified DEGs, hub genes, and TIGD1-associated pathways within the TME. These would strengthen the scientific basis for exploring TIGD1 as a potential biomarker and therapeutic target in lung cancer research.

## 5. Conclusion

Our comprehensive analysis delving into the intricate molecular landscape associated with TIGD1 expression in lung cancer has revealed significant findings. The differentially regulated genes associated with TIGD1 gene expression were identified, demonstrating their relevance to crucial molecular pathways involved in carcinogenesis, including the PI3K/AKT pathway, FOXO pathway, p53 signaling pathway, and cellular senescence. Additionally, the hub genes obtained through the intersection of DEGs with lung cancer genes, specifically CDKN2A, CTNNB1, KRAS, MYC, and TP53, exhibit multifaceted roles in key cellular processes such as cell proliferation, apoptosis, and protein metabolism. Moreover, the immune and stromal scores of hub genes further underscore their significance in the tumor microenvironment. The present study not only expands our understanding from NSCLC to lung cancer as a whole but also highlights the interconnected regulatory networks involved in carcinogenesis. The identified genes, whether as DEGs or hub genes, represent promising avenues for targeted therapeutic interventions. Their diverse roles in the intricate molecular machinery associated with TIGD1 expression provide a robust foundation for future investigations aimed at unraveling the complexities of lung cancer progression and developing effective therapeutic strategies.

Supplementary Figure Box plot representing the expression levels of TIGD1 across various cancer types from TCGA dataset. The y-axis shows the log2-transformed expression levels (TPM + 1), while the x-axis displays the different cancer types, with abbreviations corresponding to each type. Red boxes represent tumor tissues, and blue boxes represent normal tissues.

## Figures and Tables

**Table:** Log fold change (Log FC) values and adjusted p-values of the 11 identified differentially expressed genes (DEGs) in the GSE229260 dataset, indicating the differential expression characteristics within the analyzed samples.

Gene symbol	Gene name	Log FC	Adjusted p-value	Regulation
RAP1A	RAP1A, member of RAS oncogene family	−1.66	0.0016	Down
NPY1R	Neuropeptide Y receptor Y1	1.51	0.0027	Up
BTF3L4	Basic transcription factor 3-like 4	1.56	0.0010	Up
LY6K	Lymphocyte antigen 6 family member K	1.59	0.8893	Up
COL13A1	Collagen type XIII alpha 1 chain	1.61	0.0002	Up
BST2	Bone marrow stromal cell antigen 2	1.62	0.0002	Up
ZBED2	Zinc finger BED-type containing 2	1.76	0.0004	Up
RBP1	Retinol binding protein 1	2.00	0.0003	Up
SERP1	Stress associated endoplasmic reticulum protein 1	2.23	0.0001	Up
ANO1	Anoctamin 1	2.60	0.0003	Up
CDKN2A	Cyclin-dependent kinase inhibitor 2A	3.42	0.0001	Up

## Footnotes

1 Gene Expression Omnibus (2024). GEO accession display tool [online]. Website https://www.ncbi.nlm.nih.gov/geo/query/acc.cgi [accessed 15 October 2024].

2 Gene Expression Omnibus (2024). GEO accession [online]. Website https://www.ncbi.nlm.nih.gov/geo/geo2r [accessed 15 October 2024].

3 SRplot (2021-2024). SRplot [online]. Website http://www.bioinformatics.com.cn/srplot [accessed 25 September 2024].

4 STRING Consortium (2023). The Search Tool for the Retrieval of Interacting Genes/Proteins [online]. Website https://string-db.org [accessed 08 October 2024].

5 SangerBox (2023). Title [online] (in Chinese). Website http://sangerbox.com/home.html [accessed 23 September 2024].

6 University of California, UCSC Genomics Institute (2016). Xena Functional Genomics Explorer [online]. Website https://xenabrowser.net/ [accessed 17 October 2024].
