# Supplementary figures and images for: Investigating the biomarker potential and molecular targets of TIGD1 in lung cancer using bioinformatics
Source: Turk J Med Sci. 2024 Aug 19;54(6):1369–80. doi: 10.55730/1300-0144.5920 (PMC11673667; doi:10.55730/1300-0144.5920)

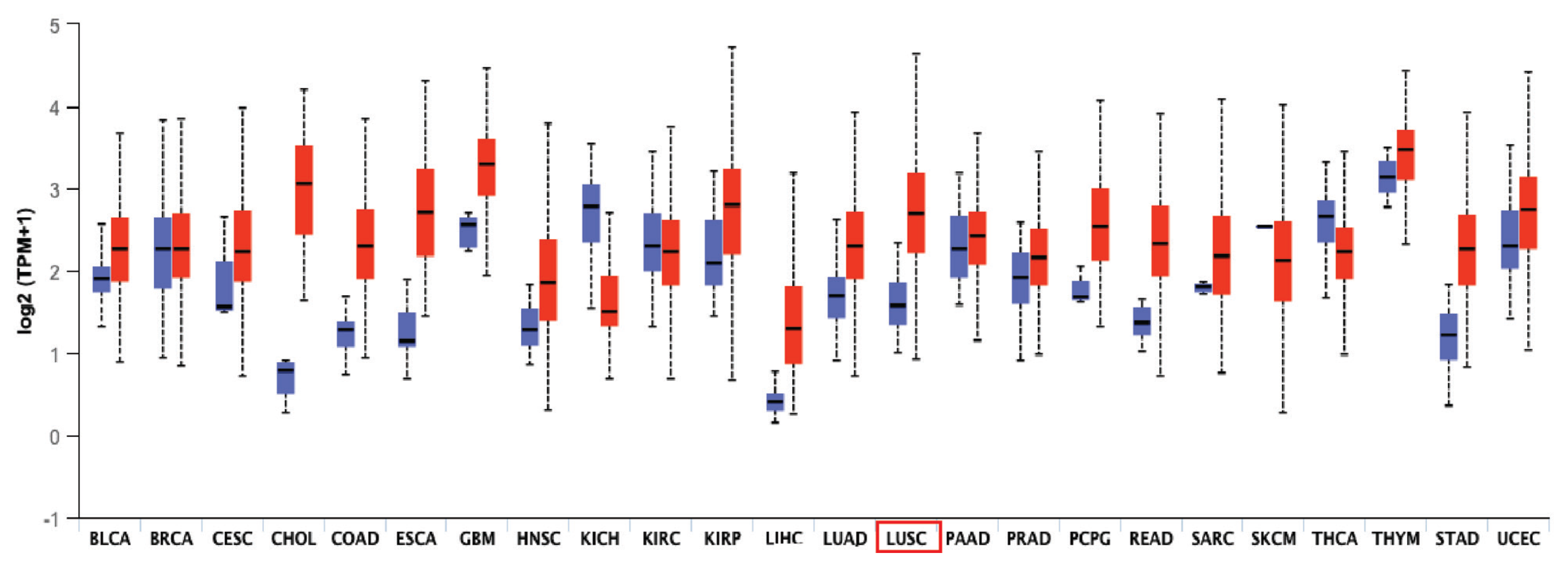

Supplement: Supplementary Figure — Box plot representing the expression levels of TIGD1 across various cancer types from TCGA dataset. The y-axis shows the log2-transformed expression levels (TPM + 1), while the x-axis displays the different cancer types, with abbreviations corresponding to each type. Red boxes represent tumor tissues, and blue boxes represent normal tissues. [file tjmed-54-06-1369s1.tif]
